# Identification of a novel HIV-1-neutralizing antibody from a CRF07_BC-infected Chinese donor

**DOI:** 10.18632/oncotarget.18594

**Published:** 2017-06-21

**Authors:** Youxiang Sun, Yuanyuan Qiao, Yuanmei Zhu, Huihui Chong, Yuxian He

**Affiliations:** ^1^ MOH Key Laboratory of Systems Biology of Pathogens, Institute of Pathogen Biology, Chinese Academy of Medical Sciences and Peking Union Medical College, Beijing 100730, China; ^2^ Center for AIDS Research, Chinese Academy of Medical Sciences and Peking Union Medical College, Beijing 100730, China

**Keywords:** HIV-1, neutralizing antibody, epitope, vaccine

## Abstract

The identification of human monoclonal antibodies (mAbs) able to neutralize a broad spectrum of primary HIV-1 isolates is highly important for understanding the immune response of HIV-1 infection and developing vaccines and therapeutics. In this study, we isolated a novel human mAb termed Y498 from a phage display antibody library constructed with the PBMC samples of a CRF07_BC-infected Chinese donor whose sera exhibited broadly neutralizing activity. Y498 cross-reacted with diverse Env antigens and neutralized 30% of 70 tested HIV-1 isolates. It efficiently blocked the binding of soluble CD4 to gp120 and competed with the CD4-binding site (CD4bs)-specific mAbs. By combining molecular docking and site-directed mutagenesis, the epitope of Y498 was characterized to contain three antigenic sites on gp120, including the CD4 binding loop in C3, the β23 in C4 and the β24-α5 in C5, which overlap the binding sites of CD4 and CD4bs-directed mAbs (b12, VRC01, A16). Therefore, Y498 is a novel neutralizing human mAb targeting a conformation-dependent CD4bs-based epitope, and its isolation and characterization could provide helpful information for elucidating human immune response to HIV-1 infection and designing effective vaccines and immunotherapeutics.

## INTRODUCTION

HIV-1 evolves with great genetic diversity, and it can be classified into distinct subtypes or clades, which pose a daunting challenge for the development of effective vaccines and immunotherapeutics; however, ∼20% of HIV-1-infected individuals do develop antibodies that broadly neutralize HIV-1 isolates. It is believed that the isolation and characterization of such broadly neutralizing antibodies (bnAbs) from different HIV-1-infected donors are critical for understanding human B cell-mediated immune response to HIV-1 infection and for designing immunogens that can elicit bnAbs by vaccination. In early 1990s, the first-generation human neutralizing antibodies (b12, 2G12, 2F5 and 4E10) were isolated from clade B-infected individuals by phage display and electrofusion or Epstein-Barr virus (EBV) transformation-based techniques [1–3]. In recent years, a number of novel neutralizing antibodies with different specificities have been isolated and characterized by using new B cell-based sorting and screening approaches [4, 5]. Notably, several conserved regions in viral envelope (Env) glycoproteins (gp120/gp41) have been frequently identified as sites of vulnerability to neutralization, which include the CD4-binding site (CD4bs), the glycan-associated V1V2 and V3 of gp120, and the membrane proximal external region (MPER) of gp41. The CD4bs on gp120 is functionally conserved and it is recognized by a number of bnAbs, including VRC01, NIH45-46, 12A12, 3BNC117, VRC-PG04, VRC-CH31, and N6, which neutralize 80%-98% of diverse HIV-1 isolates [6–9]. Some CD4bs-specific antibodies have been isolated with lower potency and breadth, such as b12, HJ16, VRC03, 1B2530, 8ANC131, A16, and DRVIA7 [1, 7, 8, 10–13].

Globally, the genetic diversity of HIV-1 is characterized by a relatively small number of genetically-defined subtypes or clades and their recombinant forms, with the subtypes A, B and C being the most prevalent viruses. In contrast, the HIV-1 epidemic in China is predominantly caused by two circulating recombinant forms (CRFs), CRF01_AE and CRF07_BC/CRF08_BC [14]. Previous studies demonstrated that recombinant strains might have enhanced fitness and pathogenicity over their parental strains, which resulted in differences in viral antigenicity [15–21]. The B/C recombinants are descendants of the parental subtype B’ from Thailand and subtype C from India, mostly in Env glycoproteins. Ma and colleagues reported that CRF07_BC strains had relatively lower net charges in the V3 loop and exclusively used CCR5 co-receptor and exhibited slow replication kinetics in primary target cells, suggesting that CRF07_BC might be superior over B’ and other subtypes in initiating infection in high-risk population [20]. Furthermore, their data also demonstrated that CRF07_BC-infected subjects developed high titers of neutralizing antibodies against heterologous strains [21]. Very recently, Hu and colleagues evaluated the prevalence, breadth and potency of neutralizing antibody responses in 98 CRF07_BC-infected subjects using a large, multi-subtype panel of Env-pseudotyped viruses, and found that top neutralizing plasmas possessed CD4bs-specific antibodies [22]. To define those neutralizing determinants, we here dedicated our efforts on the isolation and characterization of human monoclonal antibodies (mAbs) from a CRF07_BC-infected subject whose sera exhibited the most potent and broadly neutralizing activity [21]. A phage display antibody library was constructed using the peripheral blood mononuclear cells (PBMC) of subject XJ1981 and from it we isolated a novel human antibody termed Y498 (Chinese patent numbers: ZL201110077970.8 and ZL201110078167.6). In highlight, Y498 neutralized 30% of 70 tested HIV-1 isolates and targeted an epitope overlapping the CD4bs of gp120.

## RESULTS

### Isolation of a novel cross-reactive human anti-gp120 Fab

**Figure 1:**
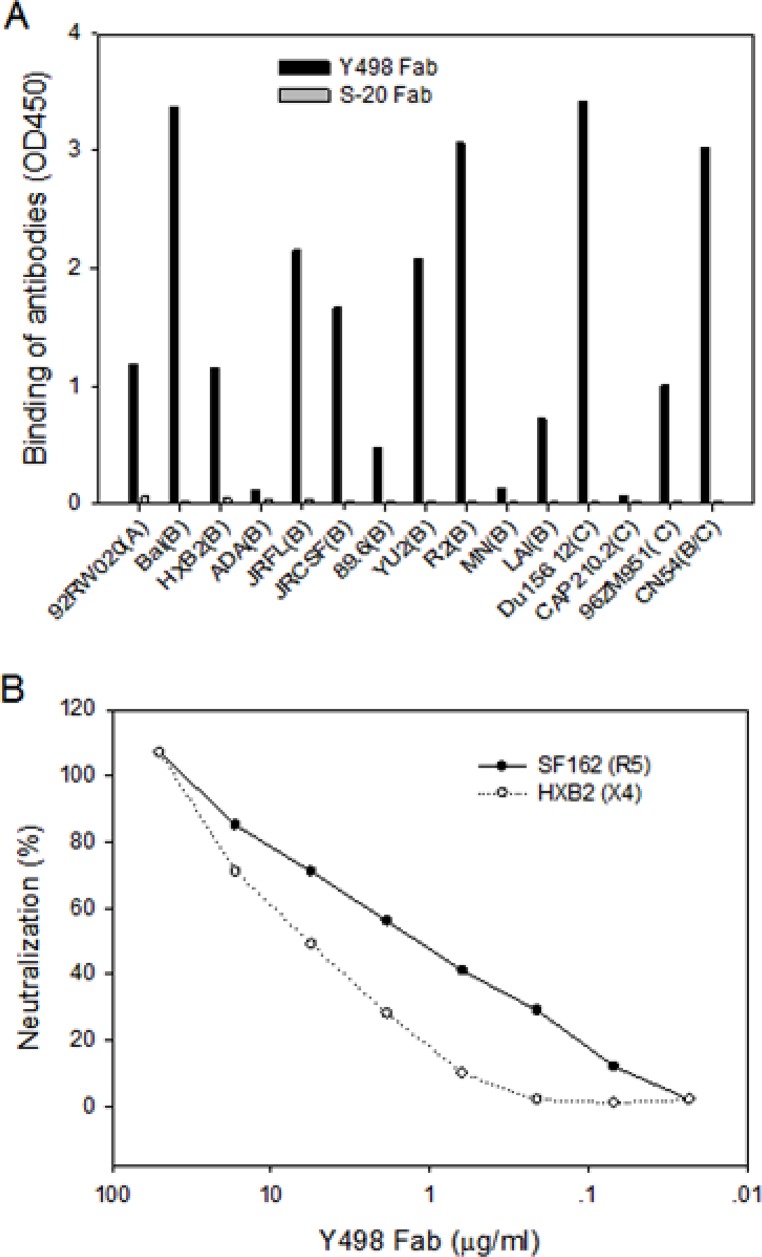
Isolation of a cross-reactive neutralizing Fab targeting gp120 **(A)** Reactivity of Y498 Fab with various rgp120 and rgp140 proteins in ELISA. 100μl of rgp120 or rgp140 (1 μg/ml) were coated to 96-well microtiter plates and 100μl supernatants of phage clone expressing Y498 Fab were tested. A human anti-SARS spike protein Fab (S-20) was used as control. **(B)** Neutralizing activity of Y498 Fab on two indicator HIV-1 pseudoviruses (SF162 and HXB2) determined by single cycle infection assay.

**Figure 2:**
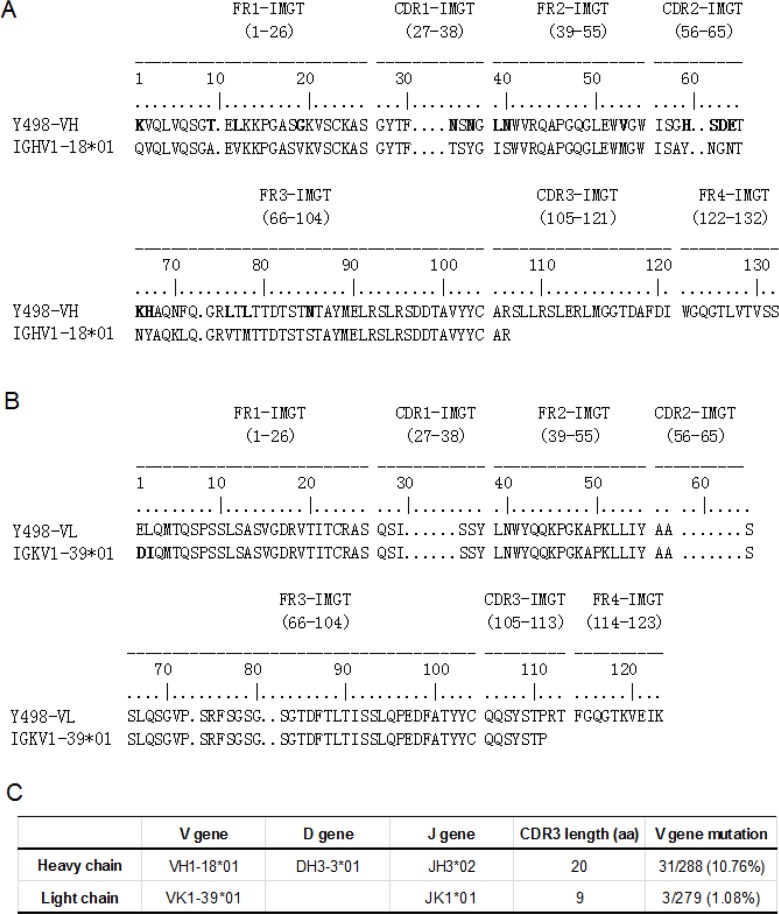
Sequence analysis of Y498 Fab **(A)** Sequence of Y498 heavy chain variable domain (VH). **(B)** Sequence of Y498 light chain variable domain (VL). **(C)** Germline, CDR3 length and the V gene mutation rate of Y498. The program IMGT/V-QUEST was applied.

A phage display Fab library was constructed using the PBMC samples of a CRF07_BC-infected subject (XJ1981), as whose sera exhibited the most potent and broadly neutralizing activity. To isolate neutralizing mAbs, we panned the library with a CRF07_BC (CN54)-derived rgp120 antigen four rounds. Among a large number of positive phages outputted, the clone Y498 showed high cross-reactive activity with various rgp120 or rgp140 proteins in ELISA (Figure 1A). Its neutralizing activity was initially evaluated with two indicator HIV-1 pseudoviruses. As shown in Figure 1B, purified Y498 Fab neutralized the R5 isolate SF162 and the X4 isolate HXB2 at a dose-dependent manner, suggesting that it was a neutralizing antibody. Sequence analysis demonstrated that the V_H_ gene of Y498 was derived from the germline IGHV-1-18*01 and its V_L_ gene was closest to the germline IGKV1-39*01 (Figure 2). Y498 was characterized by a relatively long heavy chain CDR3 loop (HCDR3) composed of 20 amino acids, a κ light chain CDR3 loop (LCDR3) containing 9 amino acids. As compared to the most of reported anti-HIV bnAbs, Y498 was also characterized by low levels of somatic hypermutations (SHM) for both the V_H_ (10.76 %) and V_L_ (1.08 %) genes.

### Y498 IgG1 is a neutralizing antibody with high binding affinity

**Figure 3:**
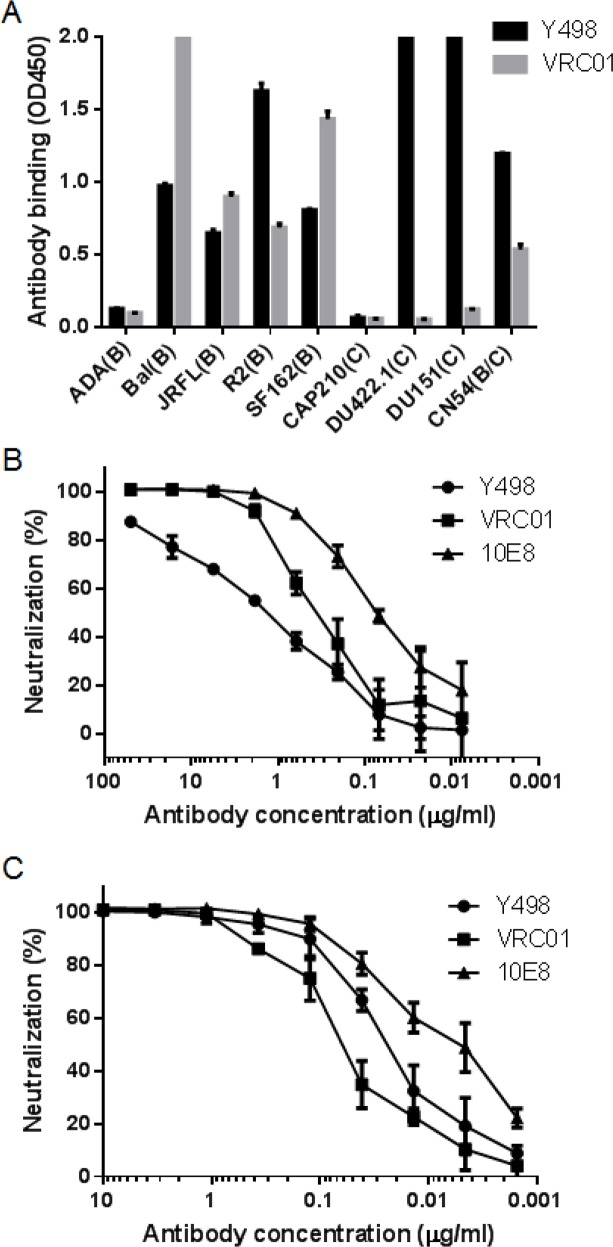
Reactivity and neutralizing activity of whole Y498 IgG1 **(A)** Reactivity of Y498 IgG1 with various Env antigens in ELISA. The neutralizing antibody VRC01 was used as a control. Neutralizing activity of Y498 IgG1 on SF162 **(B)** and NL4-3 **(C)** was determined by single cycle infection assay. The assay was performed in triplicate and repeated two times. Data are expressed as means ± standard deviations. The neutralizing antibodies VRC01 and 10E8 were used as control.

**Figure 4:**
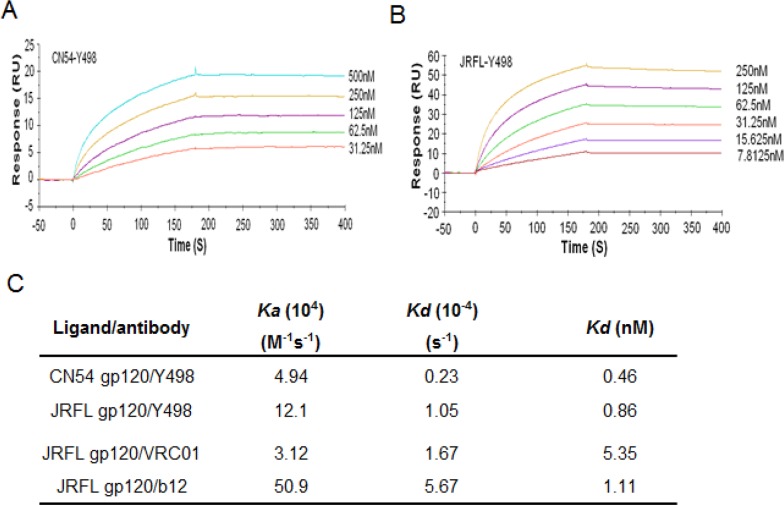
Binding affinity of Y498 IgG1 and control antibodies determined by BIACORE T200 **(A)** SPR sensorgram of Y498 IgG1 binding to CN54 gp120. The rgp120 was immobilized onto CM5 sensor chip at a concentration of 20μg/ml and Y498 was injected at concentrations of 500, 250, 125, 62.5, and 31.25 nM. **(B)** SPR sensorgram of Y498 IgG1 binding to JRFL gp120. Similarly, the rgp120 was immobilized onto CM5 sensor chip at a concentration of 20μg/ml and Y498 was injected at concentrations of 250,125, 62.5, 31.25, 15.625, and 7.8125 nM. **(C)** Binding rate constants and affinities of Y498 and control antibodies in SPR.

To facilitate its purification and characterization, we converted Y498 Fab to a full-length IgG1 format by using the pDR12 vector. Y498 IgG1 was expressed in transfected 293T cells and purified from the culture medium. Firstly, the cross-reactivity and neutralizing activity of Y498 IgG1 were tested. As shown in Figure 3, Y498 IgG1 reacted with multiple Env antigens and neutralized two indicator viruses efficiently. Next, we measured the binding affinity of Y498 by SPR assay. To do this, two representative rgp120 proteins (CN54 and JRFL) were respectively anchored onto CM5 chips. As shown in Figure 4, Y498 bound to CN54 rgp120 with a *Kd* value of 0.46 nM and bound to JRFL rgp120 with a *Kd* value at 0.86 nM, indicating it is a cross-reactive human antibody with high gp120-binding affinity.

### Neutralizing activity of Y498 on distinct subtypes of HIV-1 isolates

In view of its cross-reactivity with a number of Env antigens, we were interested to know the neutralizing spectrum of Y498 on different subtypes of HIV-1 isolates. Therefore, we assembled a panel of 70 Envs, including 3 subtype A, 16 subtype B, 6 subtype B′, 12 subtype C, 1 subtype A/C, 7 subtype A/E, 24 subtype B/C and 1 clade G. Among them, 12 Envs were recently described as a ‘global panel’ reference that represents the genetic and antigenic diversities to HIV-1 neutralizing mAbs [23]. All the 70 pseudoviruses were generated and used in single-cycle neutralization assay. As shown in Table 1, Y498 could neutralize 21 (30%) of pseudoviruses (IC_50_ ≤50 μg/ml), including 1 subtype A, 6 subtypes B and B′, 5 subtype C, 1 subtype A/E, 8 subtype B/C. As compared to b12 and VRC01, Y498 is a human mAb with limited neutralizing spectrum and potency.

**Table 1:** Neutralizing activity of Y498 and control mAbs on diverse clades of HIV-1 isolates

				IC_50_(μg/ml)^#^				
Env clone	Origin	Accession #	Clade	Tier	Y498	b12	VRC01	A16
398F1*	Tanzania	HM215312	A	1	0.19	0.08	0.08	1.85
92RW020.2	Rwanda	EU855131	A	1	>50	>50	0.33	>50
92UG037.8	Uganda	U51190	A	?	>50	>50	0.13	>50
X2278*	Spain	FJ817366	B	2	>50	>50	0.1	>50
TRO11*	Italy	AY835445	B	2	>50	>50	0.34	>50
NL4-3	USA	AY669735	B	1	0.61	0.02	0.08	0.53
SF162	USA	EU123924	B	1	0.02	0.01	0.02	0.3
JRFL	USA	AY669728	B	2	>50	0.03	0.06	50
REJO4541.67	USA	AY835449	B	2	>50	0.92	0.11	>50
RHPA4259.7	USA	AY835447	B	2	>50	0.1	0.05	37.5
PVO.4	Italy	AY835444	B	3	50	>50	1.04	>50
pCAAN5342.A2	USA	AY835452	B	2	>50	>50	1.23	>50
SC422661.8	Trinidad	AY835441	B	2	>50	0.2	0.13	>50
AC10.0	USA	AY835446	B	2	>50	36.92	2.49	>50
p1058_11.B11.1550	USA	EU289187	B	2	>50	>50	31.13	>50
pWITO4160	USA	AY835451	B	2	50	5.3	0.2	50
ADA	USA	AY426119	B	2	>50	0.69	0.14	>50
SC45.4B5.2631	Trinidad	EU289201	B	?	50	1.73	0.53	16.67
pWEAUd15.410.5017	USA	EU289202	B	?	>50	1.81	0.22	>50
B01	China	ABY71518	B′	?	>50	>50	2.12	>50
B02	China	ABY71519	B′	?	>50	0.22	0.07	33.13
B04	China	ABY71521	B′	?	5.69	>50	31.13	>50
CNE4	China	HM215413	B	?	>50	>50	0.83	11.02
CNE11	China	HM215398	B′	?	>50	1.58	0.23	>50
CNE14	China	HM215400	B′	?	>50	2.21	0.56	>50
25710*	India	EF117271	C	1	50	>50	0.36	>50
CE0217*	Malawi	FJ443575	C	3	>50	18.22	0.32	>50
CE1176*	Malawi	FJ444437	C	2	>50	>50	1.83	>50
DU422.1	South Africa	DQ411854	C	2	50	0.2	>50	>50
ZM214M.PL15	Zambia	DQ388516	C	2	>50	>50	2.64	>50
CAP210.2.00.E8	South Africa	DQ435683	C	2	50	>50	>50	>50
CAP45.2.00.E8	Durban	DQ435682	C	2	>50	2.3	8.95	>50
ZM109F.PB4	Zambia	AY424138	C	1	>50	>50	0.11	>50
CNE2	China	HQ699950.1	C	?	9.08	0.45	0.05	>50
CNE17	China	HM215303	C	?	14.22	>50	1.27	42.86
CNE58	China	HM215421.1	C	?	>50	>50	0.14	50
CNE65	China	HQ699980.1	C	?	>50	>50	0.96	>50
246F3*	Tanzania	HM215279	A/C	2	>50	>50	0.41	>50
CNE3	China	HM215410	A/E	?	>50	>50	4.94	>50
CNE8*	China	HM215427	A/E	2	>50	>50	0.43	>50
CNE55*	China	HM215418	A/E	3	>50	>50	0.42	>50
CNE59	China	HM215422.1	A/E	?	>50	>50	34.15	50
YN192.31	China	GU475046	A/E	?	>50	>50	0.32	>50
GX2010.36	China	ADD83183	A/E	?	50	>50	3.22	26.93
AE03	China	EU363851.1	A/E	?	>50	>50	0.01	>50
BJOX2000*	China	HM215364	B/C	2	>50	>50	>50	>50
CH119*	China	EF117261	B/C	2	36.47	>50	0.97	5.56
XJ16-6	China	HQ326133.1	B/C	?	>50	10.75	3.5	>50
HB5-3	China	ADT65143	B/C	2	>50	>50	0.27	>50
SC19-15	China	ADT65148	B/C	?	>50	26.59	0.19	50
YN148r-9	China	ADT65158	B/C	?	>50	1.27	2.02	>50
BC07	China	ABY71530	B/C	?	>50	>50	0.18	>50
CNE7	China	HM215426	B/C	?	>50	2.25	0.56	>50
CNE15	China	HM215401	B/C	?	>50	12.75	0.07	>50
CNE16	China	HM215402	B/C	?	>50	9.62	0.14	>50
CNE23	China	HM215408	B/C	?	>50	5.06	16.29	>50
CNE30	China	HM215411	B/C	?	50	21.83	0.59	>50
CNE40	China	HM215414	B/C	?	0.07	>50	0.3	0.26
CNE46	China	HQ699971	B/C	?	>50	24.63	3.03	>50
CNE47	China	HQ699972	B/C	?	>50	>50	29.02	>50
CNE49	China	HQ699974	B/C	?	>50	>50	0.31	>50
CNE53	China	HM215417	B/C	?	32.73	>50	0.11	14.51
CH064.20	China	EF117254	B/C	2	>50	19.28	0.55	50
CH070.1	China	EF117255	B/C	3	>50	>50	10.76	50
CH091.9	China	EF117256	B/C	2	6.7	>50	0.23	5.83
CH110.2	China	EF117257	B/C	?	>50	10.8	4.16	>50
CH114.8	China	EF117264	B/C	3	31.6	>50	0.29	45.3
CH117.4	China	EF117262	B/C	2	50	>50	0.11	50
CH120.6	China	EF117260	B/C	3	50	>50	4.5	50
X1632*	Spain	FJ817370	G	2	>50	>50	0.29	>50
% of neutralization					30%	42.86%	95.71%	32.86%

* represents a ‘global panel’ HIV-1 isolates (reference 29); ? means ‘not determined’;

^#^ indicates that the IC_50_ values of three control mAbs were obtained from a previous study (reference 13). The neutralizing IC_50_ values are colored to show the strength (10-50 = green; 1-10 = light green; 0.1-1= yellow; .01-.1= red).

### Y498 targets an epitope overlapping the CD4 binding site of gp120

**Figure 5:**
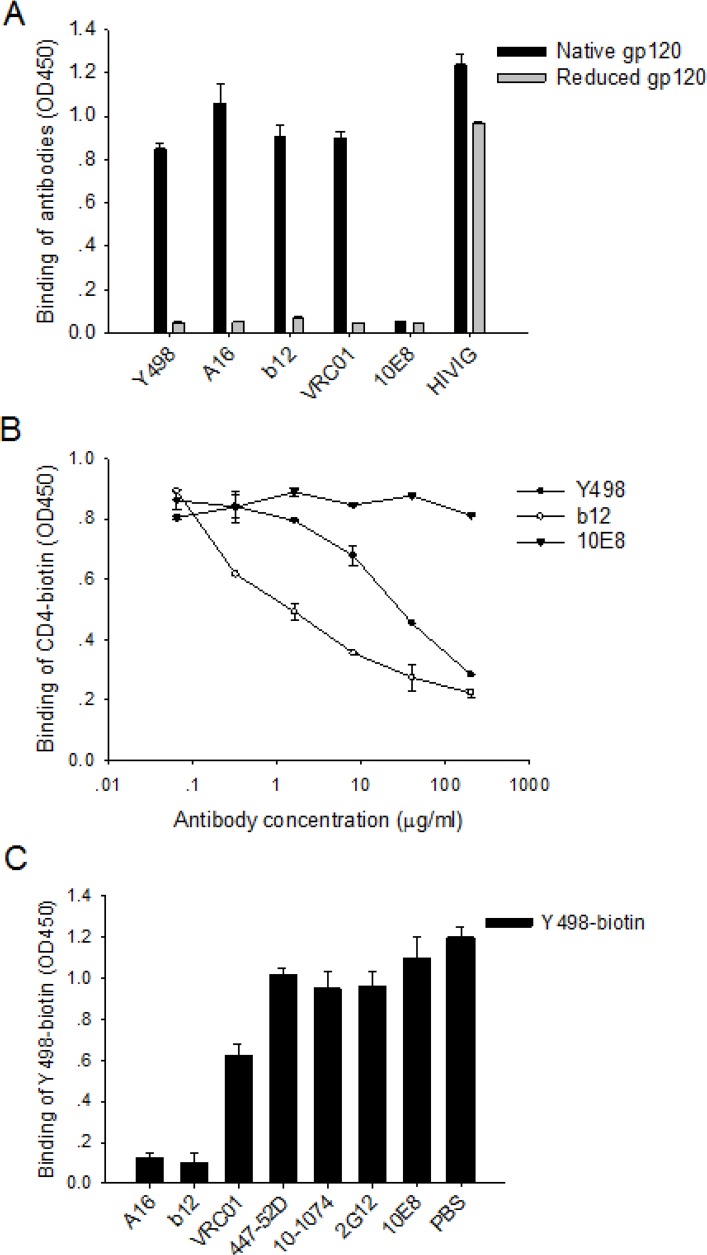
Y498 targets a conformational epitope overlapping the CD4bs of gp120 **(A)** Reactivity of Y498 and control antibodies with DTT-reduced gp120 in ELISA. **(B)** Inhibition of Y498 and control antibodies on the binding of biotinylated sCD4 to gp120 in ELISA. **(C)** Inhibition of competitive anti-Env antibodies on the binding of biotinylated Y498 to gp120 in ELISA. The assay was performed in duplicate and repeated two times. Data are expressed as means ± standard deviations.

To define the neutralizing epitope of Y498, we firstly compared its reactivity with native and DTT-reduced rgp120. As shown in Figure 5A, Y498 reacted strongly with the native but not the reduced rgp120, similar to three CD4bs-directed human mAbs (A16, b12 and VRC01). As control, the polyclonal antibody HIVIG reacted with each of the rgp120 proteins, whereas the gp41-specific antobody10E8 had no reaction. The results suggested that Y498 targets a conformation-dependent epitope on gp120.

To further localize its epitope, we investigated whether Y498 blocked the interaction between rgp120 and the receptor CD4. To this end, soluble CD4 (sCD4) was labeled by biotin and used in a competition ELISA assay. As shown in Figure 5B, Y498 and b12 could efficiently inhibit the binding of biotinylated sCD4 to the coated rgp120 at a dose-dependent manner, while an anti-gp41 control mAb (10E8) exhibited no such activity, suggesting that Y498 recognized a gp120 epitope overlapping the CD4bs. To verify this, we further performed a competition assay to determine whether biotinylated Y498 (Y498-biotin) could be blocked by three CD4-directed mAbs (A16, b12, VRC01), while several anti-Env mAbs (10-1074, 447-52D, 2G12, 10E8) were used as experimental control. As shown in Figure 5C, A16 and b12 could efficiently block the binding of Y498-biotin to rgp120, while VRC01 worked partially. As expected, two anti-V3 mAbs (10-1074 and 447-52D), the glycan-dependent anti-gp120 mAb 2G12 and gp41 MPER-specific mAb 10E8 exhibited no blocking function. Meanwhile, we also measured the binding affinity of b12 and VRC01 by SPR and the results were presented in Figure 4C. It was found that the *Kd* value of VRC01 to JRFL rgp120 was significantly lower than that of b12 and Y498, implying its weaker competitive ability.

### Homology modeling and molecular docking of Y498

**Figure 6:**
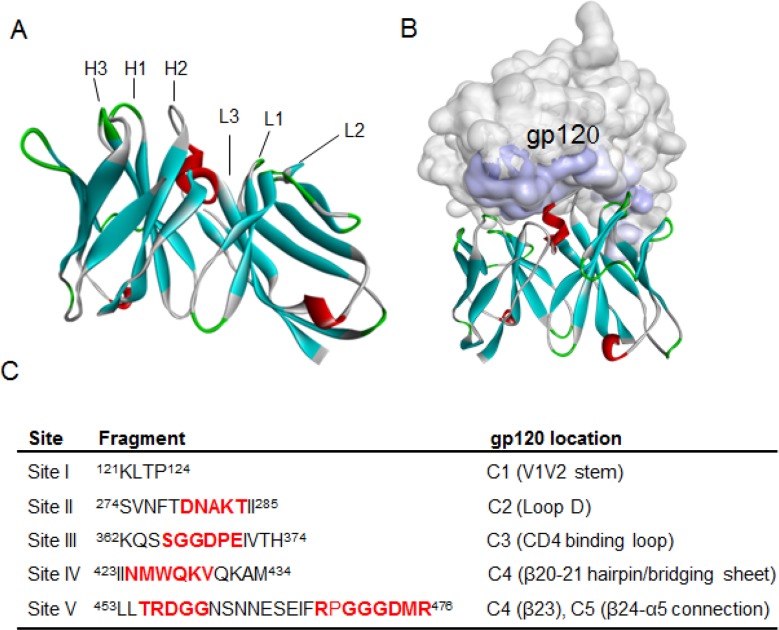
Homology modeling and molecular docking of Y498 by Discovery Studio 3.5 program **(A)** Homology modeling of Y498. Heavy chain and light chain and a combination of heavy/light chain from antibodies with PDB codes 2XQB_H, 1HEZ_A and 2XTJ_BD were used as templates for structure alignments to produce Y498 homology model. The CDR loops in the Y498 VH and VL domains are labeled. **(B)** Molecular docking of Y498. The 3D structure of gp120 (3DNN) was retrieved from the PDB and initialized as a receptor molecule with the Protein Preparation tool in the program. Gp120 is labeled in gray, Y498 is labeled in cyan, and the contact zone of gp120 is labeled in slate. **(C)** Contact fragments and their locations on gp120 were identified by molecular docking. The residues critical for CD4 binding are labeled in red.

To gain more insights into the Y498 epitope, we performed a molecular docking analysis to predict the interaction of Y498 and rgp120. First, the BLAST search revealed that Y498 had amino acid similarity with three PDB entries (2XQB_H, 1HEZ_A, 2XTJ_BD), and then a homology model was produced by the Modeler block program of Discovery Studio 3.5 (Figure 6A). While Y498 was docked onto the 3D structure of gp120, five interacting fragments in different conserved gp120 domains were localized (Figure 6B-6C), including ^121^KLTP^124^ in the V1V2 stem of C1 (site I), ^274^ SVNFTDNAKTII^285^ in the loop D of C2 (site II), ^362^KQSSGGDPEIVTH^374^ in the CD4-binding loop of C3 (site III), ^423^IINMWQKVQKAM^434^ in the β20-21 hairpin (bridging sheet) of C4 (site IV), ^453^LLTRDGGNSNNESEIFRPGGGDMR^476^ in the β23 of C4 and the β24-α5 of C5 (site V). Obviously, these sites contain a number of critical residues of CD4bs, which verified the Y498 epitope and its neutralizing function.

### Characterization of the Y498 epitope residues by mutagenesis

**Figure 7:**
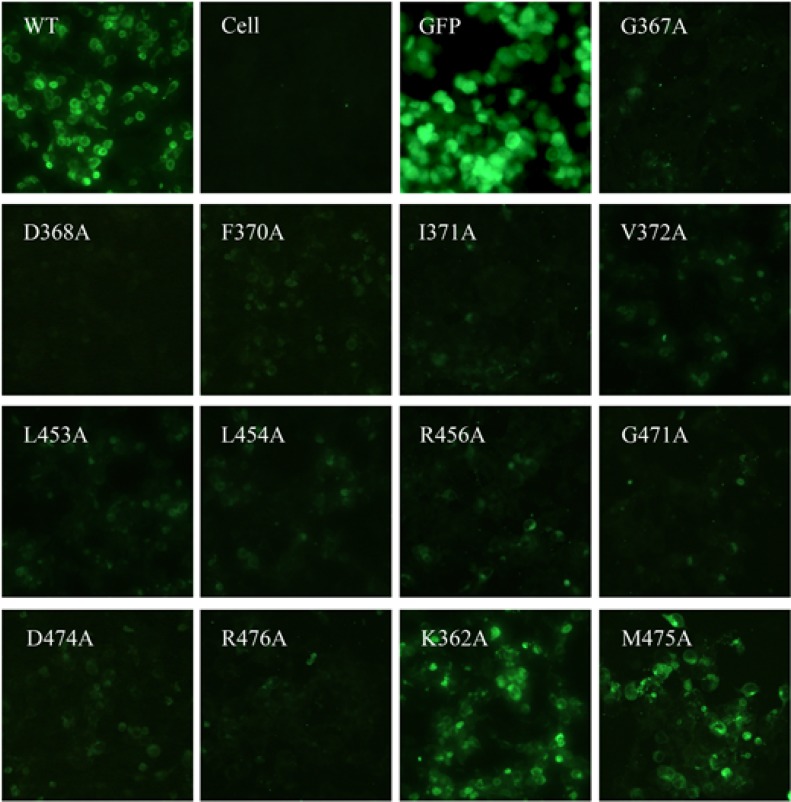
Binding activity of Y498 with the wild-type (WT) and mutant HIV-1 Envs determined by IFA The plasmids encoding Envs were transiently expressed in 293T cells by transfection. Y498 was tested at a concentration of 20μg/ml and detected by a DyLight®488 labeled-rabbit anti-human antibody. The experiments were repeated three times and the representative data are shown. The amino acid numbering of Env is based on the HIV-1 HXB2 sequence.

**Figure 8:**
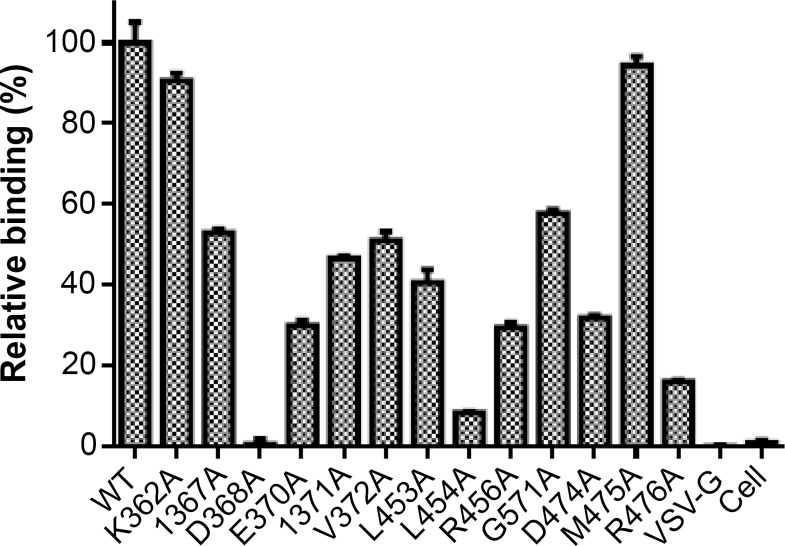
Relative reactivity of Y498 with the Env-expressing cell lysates determined by capture ELISA A sheep anti-gp120 antibody (D7324) was coated in ELISA wells as a capture and Y498 was used at 2 μg/ml to probe the WT or mutant Env in the protein extractions of transfected cells. The experiments were repeated three times and the data are expressed as means ± standard deviations.

To map the binding epitope of Y498, we generated a panel of 61 Env mutants that carry single amino acid substitutions corresponding to the predicted residues by molecular docking. Three residues (I284, I285, F468) were excluded because they were not exposed on the surface of gp120. All the residues were mutated to alanine except two naturally-occurring alanines (A281 and A433), which were changed to a glycine. The wild-type (WT) and mutated Envs were expressed in 293T cells by transfection, and the reactivity of Y498 was firstly detected by IFA. As shown in Figure 7, the substitutions of eleven residues could markedly reduce the binding activity of Y498, including 5 residues in the CD4 binding loop (G367, D368, E370, I371, V372), 3 residues in the β23 strand (L453, L454, R456), and 3 residues in the β24-α5 connection site (G471, D474, R476). We also determined the reactivity of Y498 with the cell lysates by capture ELISA. Consistent to the IFA results, the same panel of residues was found to be critical for the binding by Y498 (Figure 8). In contrast, the substitutions of other 50 residues had no or minor effects on Y498's reactivity in both IFA and capture ELISA experiments (data not shown), suggesting that they might not be involved in the epitopic composition. Therefore, the results suggested that three discontinuous sites on gp120 (CD4 binding loop, β23 strand and β24-α5 connection) critically determine the Y498 epitope.

### Comparison of the Y498 epitope with the binding sites of CD4 and neutralizing mAbs

**Figure 9:**
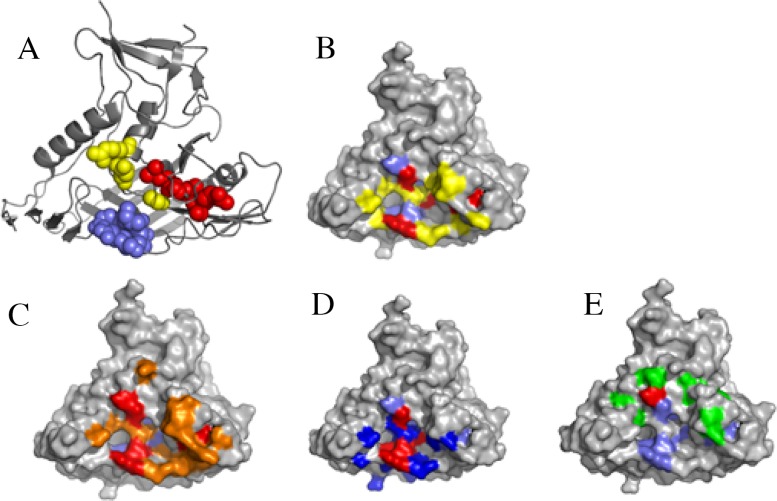
Analysis of Y498 epitope by surface representation **(A)** Presentation of the Y498 epitope on gp120 core (PDB ID: 3DNN). The residues in the CD4-binding loop (G367, D368, E370, I371, V372) are labeled in slate, the residues in the β23 strand (L453, L454, R456) are labeled in red, and the residues in the β24∼α25 connection region (G471, D474, R476) are labeled in yellow. **(B)** The overlapping residues of Y498 epitope and CD4bs. The Y498-binding residues are marked in slate, the CD4-binding residues are in yellow, and their overlapping residues (G367, D368, R456, G471, D474) are in red. **(C)** Comparison of the Y498 and b12 epitopes. The Y498-binding residues are marked in slate, the b12-binding residues are in orange, and their overlapping residues (G367, D368, E370, I371, G471, D474) are in red. **(D)** Comparison of the Y498 and VRC01 epitopes. The Y498-binding residues are marked in slate, the VRC01-binding residues are in blue, and their overlapping residues (G367, D368, R456, G474) are in red. **(E)** Comparison of the Y498 and A16 epitopes. The Y498-binding residues are marked in slate, the A16-binding residues are in green, and their overlapping residue (R476) is shown in red.

To further understand the mechanism of Y498-mediated neutralization, we presented its potential epitope residues on the 3-dimensional structure (3D) of gp120 protein (PDB No: 3DNN) by the program PyMol. As shown in Figure 9, three antigenic sites are adjacent to each other in the 3D structure, and apparently, Y498 targets an epitope overlapping with the binding sites of the receptor CD4 and three CD4bs-directed neutralizing mAbs (b12, VRC01, A16). Specifically, the Y498 epitope respectively shares five residues with the footprint of CD4 binding (G367, D368, R456, G471, D474), six residues with the b12 epitope (G367, D368, E370, I371, G471, D474), four residues with the VRC01 epitope (G367, D368, R456, G474), and one residue with the A16 epitope (R476).

## DISCUSSION

In the present study, we identified a novel HIV-1 neutralizing human antibody by panning a phage display Fab library, which was constructed with the PBMC sample of a CRF07_BC-infected Chinese donor whose sera exhibited broadly neutralizing activity. Y498 was genetically characterized with the unique VH and VL sequences, a relatively long heavy chain CDR3 loop (HCDR3), and low levels of somatic hypermutaions (SHM). Y498 functionally neutralized 30% of diverse HIV-1 isolates that represent a global antigenic diversity and competed with CD4 and CD4-binding site specific mAbs for gp120 binding. The epitope of Y498 was mapped to the CD4-binding loop, β23 strand and β24-α5 connection region, which overlap with the binding sites of CD4 and neutralizing antibodies (VRC01, b12, and A16).

To infect target cells, HIV-1 sequentially binds to the cellular receptor CD4 and a coreceptor (CCR5 or CXCR4) and then fuses its membrane with the cell membrane. The viral Env glycoproteins are composed of the surface subunit gp120 and the transmembrane subunit gp41, which not only mediate the receptor binding and membrane fusion but also serve as the major targets of neutralizing antibodies. Previous studies have demonstrated that both of gp120 and gp41 contain antigenic sites targeted by broadly neutralizing antibodies (bnAbs), such as the glycan-associated V1V2 and V3, the conformation-dependent CD4bs of gp120, the MPER and fusion peptide of gp41, and the conserved regions stretching across two subunits [24–28]. Predominantly, the CD4bs-directed neutralizing antibodies were frequently identified from donors whose sera possess potent and broadly neutralizing activity [1, 7–9, 11, 29]. As one of the first-generation of bnAbs, the CD4bs-directed antibody b12 was isolated in early 1994 from a clade B-infected long-term non-progressor (LTNP) by phage display technology [1]. In early 2010, the second anti-CD4bs bnAb HJ16 was isolated with improved EBV immortalization and culture methodologies, which showed a similar neutralizing spectrum with b12 (∼40%) [12]. However, a number of novel CD4bs-specific bnAbs were found by applying advanced technologies [7, 8, 26, 28]. By applying a resurfaced stabilized gp120 core protein (RSC3) as a bait and single B cell PCR amplification of antibody sequences, Wu and colleagues discovered two new CD4bs-specific bnAbs, VRC01 (∼90% breadth) and VRC03 (∼50% breadth) [8]. While the RSC3-bait strategy was used on two additional donors, several potent anti-CD4bs bnAbs, including VRC-PG04 and VRC-CH31 (∼80% breadth) were fished out [6]. By using new PCR primers and modified gp140/gp120 probes, Scheid et al identified a number of anti-CD4bs antibodies that were both potent and broadly neutralizing (∼90% breadth), including NIH45-46, 3BNC117, and 12A12 [7]. Very recently, we isolated a CD4bs-specific antibody named A16 by sequentially panning the phage display antibody library with distinct clades of Env glycoproteins [10], while Kong and colleagues isolated an additional antibody (DRVIA7) from a donor in the same B’-infected cohort of former plasma donors [11]. Both A16 and DRVIA7 neutralized HIV-1 isolates with somewhat less potency and breadth, similar to that of b12 and HJ16.

Broadly and potent neutralizing antibodies are usually found in long-term none progressing HIV-1-infected subjects and usually feature extraordinary long CDR3 regions and high SHM rates [8, 9, 30, 31], which imply a complicated experience during antibody affinity maturation *in vivo* [28, 32]. In comparison to other eminent CD4bs-directed antibodies such as VRC01 and N6 [9, 28, 33], Y498 owns a relatively low level SHM (10.8% in VH and 1.1% in VL), similar to that of the DRVIA7 family mAbs [11]. One may speculate that Y498 needs a rather longer period to accomplish affinity maturation by somatic mutations, and that the existence of steric hindrance formed by the variable loops and glycan on gp120 may shield the access of Y498 thereby restricting its neutralizing range [11, 34, 35].

The CD4bs of the HIV-1 Env spike is functionally conserved and it has thus been considered as an ideal template for designing vaccine immunogen and therapeutic approaches [26, 28, 29]. It is highly necessary to isolate and characterize new neutralizing antibodies targeting this site, as they would help our understanding on human anti-HIV immune responses and thus facilitate the development of effective vaccines and immuotherapeutic strategies. Actually, Y498 was initially isolated in the early 2009 and a Chinese patent was filed in 2011. Due to the lack of viral panels and structural platforms, we suffered from its functional and epitopic analyses over past years. As it is the first human neutralizing antibody isolated from a CRF07_BC-infected donor and possesses the unique VH and VL sequences, neutralizing specificity and binding epitope, we will definitely continue our efforts to characterize its biochemical and immunological characteristics, and hopefully a crystal structure for the Y498/gp120 complex would be available that can elucidate its neutralizing mechanism in detail.

## MATERIALS AND METHODS

### Cells and reagents

293T cells were purchased from the American type culture collection (ATCC). TZM-bl indicator cells stably expressing large amounts of CD4 and CCR5 along with endogenously expressed CXCR4, HIV-1 Env panels (clades A, B, B′, C, A/E and B/C), plasmids expressing human mAbs (b12, VRC01, 10E8 and 10-1074), and purified human mAbs 447-52D and 2G12 and polyclonal antibody HIVIG were obtained through the AIDS Reagent Program, Division of AIDS, NIAID, NIH. Recombinant rgp120 or rgp140 derived from diverse subtypes of HIV-1 isolates (92RW020, Bal, HXB2, ADA, JRFL, JRCSF, 89.6, R2, YU2, MN, LAI, Du151, DU156.12, Du422.1, CAP210.2, 96ZM951, SF162 and CN54) were purchased from the Immune Technology Corp (New York, NY) or Enzyme LLC (Gaithersburg, MD). The control mAbs A16 (anti-gp120) and S-20 (anti-SARS spike protein) were prepared in our laboratory.

### Ethics and human subjects

This study was reviewed and approved by the ethics committee of the Institute of Pathogen Biology, Chinese Academy of Medical Sciences and Peking Union Medical College. All methods were performed in accordance with the relevant guidelines and regulations. Human peripheral blood mononuclear cells (PBMC), which were provided by Dr. Yiming Shao at the Chinese Center for Disease Control and Prevention, were collected from the subject XJ1981 with informed consent and were a leftover from the previous studies [20, 21].

### Construction, panning and screening of phage Fab library

A phage display Fab library was constructed, panned and screened as described previously [1]. In brief, mRNA was purified from the PBMC of subject XJ1981 and cDNA was transcribed. The heavy (H) and light (L) chain genes were amplified from the cDNA by PCR and then cloned into the phagemid pComb3XSS vector (kindly provided by Dr. Carlos Barbas at the Scripps Research Institute, La Jolla, CA). The anti-HIV phage display library was assembled and characterized. To isolate specific antibodies, the library was panned with CRF07_BC (CN54)-derived gp120 antigen. After four rounds of panning, phage clones were screened for binding to CN54 gp120 by phage ELISA. The VH and VL of the selected clones were sequenced and analyzed by the program IMGT/V-QUEST (http://imgt.org). Soluble Fab was expressed in *E. coli* and purified by Ni-NTA Super-flow columns.

### Conversion of Fab to IgG1 and IgG1 expression

The Fab heavy and light chains were amplified and recloned in pDR12 vector (kindly provided by Dr. Dennis R. Burton at the Scripps Research Institute) for whole IgG1 expression. The resulting construct was verified by DNA sequencing. The IgG1 antibodies were expressed in 293T cells by transient transfection and purified from the culture medium by protein A/G sepharose 4 fast flow following the protocol from the manufacturer (GE Healthcare).

### Direct enzyme-linked immunosorbent assays (ELISA)

Reactivity of Fab or IgG antibodies with various Env antigens was measured by ELISA. Briefly, 1 μg/ml rgp120 or rgp140 was used to coat 96-well microtiter plates (Costar, Corning, NY) in 0.1 M carbonate buffer (pH 9.6) at 4°C overnight. The plates were blocked with 3% bovine serum albumin (BSA) for 1 h at RT and then washed with PBS-0.05% Tween 20 (PBST). A tested antibody was added into wells and incubated at 37°C for 1 h, followed by three washes with PBST. Bound antibodies were detected with HRP-conjugated goat anti-human IgG (Sigma, Aldrich) at 37°C for 1 h, followed by three washes. The reaction was visualized by addition of 3,3^’^,5,5′-tetramethylbenzidine (TMB) substrate (Sigma), and stopped by the addition of 2 M H_2_SO_4_. The absorbance at 450 nm was measured by an ELISA plate reader (Bio-Rad).

To determine the effect of disulfide bond reduction on the binding of antibodies, a rgp120 (R2) antigen at a concentration of 1 μg/ml was treated with 10 mM dithiothreitol (DTT) for 1 h at 37°C and then terminated with 50 mM iodoacetamide for 1 h at 37°C. Both native and reduced rgp120 antigens were coated in the wells of microtiter plates and a standard ELISA was performed as described above.

### Competition ELISA

To determine the inhibitory ability of the newly-isolated antibody Y498 and control anti-gp120 antibodies (b12 and 10E8) on the binding of gp120 and CD4 receptor, a competition ELISA was performed as described previously [10]. Briefly, sCD4 was biotinylated using an EZ-Link NHS-PEO solid phase biotinylation kit (Thermo Fisher Scientific, CA), and then added to the rgp120 (R2)-coated ELISA wells in the presence or absence of diluted competitive antibodies. Following the incubation at 37°C for 1 h and extensive washing, the biotinylated sCD4 bound to rgp120 was detected by HRP-conjugated streptavidin (Sigma). Similarly, Y498 was biotinylated and its competition with the un-biotinylated anti-gp120/gp41 antibodies (A16, b12, VRC01, 447-52D, 2G12, and 10E8) was determined.

### Neutralization assays

The neutralizing activity of antibodies was measured by single cycle infection assay as described previously [36]. Briefly, HIV-1 pseudovirus was generated via cotransfection of 293T cells with an Env-expressing plasmid and a backbone plasmid pSG3^Δenv^ that encoded Env-defective, luciferase-expressing HIV-1 genome. Supernatants were harvested 48 h after transfection and the infectivity of pseudoviruses was titrated in TZM-bl cells. An antibody was prepared in 3-fold dilutions and incubated with a tested virus at 37°C for 1h. The mixture was added to TZM-bl cells (10^4^/well) in triplicate and incubated for additional 48-72 h at 37°C. Supernatants were discarded and the cells were lysed, followed by quantitation of the luciferase activity using luciferase assay reagents and a Luminescence Counter (Promega, Madison, Wisconsin, USA). The percentage inhibition and IC50 values were calculated using Graphpad prism-6 software.

### Affinity determination by surface plasmon resonance (SPR)

The binding kinetics of human mAbs (Y498, VRC01, b12) with rgp120 antigens (CN54, JRFL) were analyzed by surface plasmon resonance (SPR) using a BIACORE T200 instrument (GE Healthcare). Briefly, rgp120 was covalently immobilized onto a sensor chip (CM5) using standard amine coupling chemistry. An antibody at two-fold dilutions was injected over the channels at a flow speed of 30 μl/min for 3 minutes and allowed to dissociate for another 10-15 minutes before regeneration with 25 μl injections of 10mM glycine-HCl (pH2.5) at a flow rate of 30 μl/min. Sensorgrams were corrected with appropriate blank references and fitted globally with Biacore Evaluation software using a 1:1 Langmuir model of binding.

### Homology modeling and molecular docking of Y498

Discovery Studio 3.5 (DS 3.5, Accelrys Software Inc., San Diego, CA) Modeler block was used to construct a homology model of Y498. Heavy chain and light chain and a combination of heavy/light chain from antibodies with PDB codes 2XQB_H, 1HEZ_A and 2XTJ_BD were used as templates for structure alignments to produce Y498 homology models [23]. The CDR loops in the Y498 VH and VL domains were identified, refined and reconstructed using the Model Antibody Loops protocol and the Loops refinement protocol in DS3.5. Ramachandran plots and the Verify protein (Profiles-3D) protocol were used to validate the final VH and VL model. For protein-ligand docking, 3D structure of gp120 (PDB: 3DNN) was retrieved from the PDB and initialized as a receptor molecule with the Protein Preparation tool. All possible torsion angles in Y498 ligand molecules were set to rotate freely. The flexible molecular docking of the Y498 variable domain onto the gp120 model was accomplished using the ZDOCK protocol in DS 3.5 with high predictive accuracy Generic Algorithm (GA) parameters. The complex of ligand and Y498 Fab were optimized by RDock. Finally, the protein-protein interactions were evaluated by E_RDock, and the top ranked ligands were obtained.

### Site-directed mutagenesis

A panel of Env mutants was generated using a site-directed mutagenesis kit (Agilent, Santa Clara, CA) as described previously [10]. The plasmid encoding Env glycoprotein (gp160) of HIV-1 NL4-3 was used as a template. The primers for introducing mutations were designed and synthesized according to the manufacturer's instructions. The PCR products were digested with DpnI to remove the methylated parental DNA, and DNAs containing the desired mutations were transformed into competent cells. The introduced mutations were verified by DNA sequencing and the expression of Envs was confirmed by Western-blot and capture ELISA. The functionality of Env mutants were confirmed by pseudotyped viruses in single cycle infection assay as described above.

### Immunofluorescence assay (IFA)

The reactivity of Y498 with the wild-type (WT) and mutant Envs was measured by IFA as described previously [10]. Briefly, 293T cells (2 × 10^5^) were seeded in 24-well plates and incubated for 12 h. The plasmids encoding Envs were transfected into 293T cells and incubated for additional 24-36 h. The cells were then immobilized with 4% paraformaldehyde and permeabilized with 0.25% Triton-X-100 (Sigma), followed by blocking with 5% BSA at RT. After washing twice with PBS, Y498 was added to the cells at a final concentration of 20 μg/ml and incubated at 4°C overnight. After 3 times of washes, bound antibodies were probed by DyLight®488 labeled-rabbit anti-human antibody (Abcam, Cambridge, MA) and the images were captured under an immunofluorescence microscope.

### Capture ELISA

The reactivity of Y498 with the wild-type (WT) and mutant Envs was also measured by capture ELISA. In brief, an affinity-purified sheep anti-gp120 antibody (D7324) (Aalto Bio Reagents, Dublin, Ireland) was coated in ELISA wells and incubated at 4°C overnight. After blocking and washing, diluted protein extractions from the cells transfected with Env-expressing plasmids or mock plasmid were added and incubated at 37°C for 1 h. After 5 washing, Y498 was added at 2 μg/ml and incubated for 1 h. After washing, the bound antibodies were detected with HRP-conjugated goat anti-human IgG (Sigma). The results were visualized as described in direct ELISA.

## Abbreviations

HIV-1: human immunodeficiency virus type 1
mAb: monoclonal antibody
CD4bs: CD4-binding site
PBMC: peripheral blood mononuclear cell
HCDR3: heavy chain CDR3 loop
SHM: somatic hypermutaion
ELISA: enzyme-linked immunosorbent assay
SPR: surface plasmon resonance
IFA: immunofluorescence assay
